# Simulation for Supporting Scale-Up of a Fluidized Bed Reactor for Advanced Water Oxidation

**DOI:** 10.1155/2014/348974

**Published:** 2014-09-17

**Authors:** Farhana Tisa, Abdul Aziz Abdul Raman, Wan Mohd Ashri Wan Daud

**Affiliations:** Department of Chemical Engineering, Faculty of Engineering, University of Malaya, 50603 Kuala Lumpur, Malaysia

## Abstract

Simulation of fluidized bed reactor (FBR) was accomplished for treating wastewater using Fenton reaction, which is an advanced oxidation process (AOP). The simulation was performed to determine characteristics of FBR performance, concentration profile of the contaminants, and various prominent hydrodynamic properties (e.g., Reynolds number, velocity, and pressure) in the reactor. Simulation was implemented for 2.8 L working volume using hydrodynamic correlations, continuous equation, and simplified kinetic information for phenols degradation as a model. The simulation shows that, by using Fe^3+^ and Fe^2+^ mixtures as catalyst, TOC degradation up to 45% was achieved for contaminant range of 40–90 mg/L within 60 min. The concentration profiles and hydrodynamic characteristics were also generated. A subsequent scale-up study was also conducted using similitude method. The analysis shows that up to 10 L working volume, the models developed are applicable. The study proves that, using appropriate modeling and simulation, data can be predicted for designing and operating FBR for wastewater treatment.

## 1. Introduction

Advanced oxidation processes (AOPs) have emerged to be one of the alternatives for treating effluents containing very toxic organic compounds [1]. The use of advanced oxidation processes in treating wastewater containing refractory and inhibitory organics has attained a good recognition over the past few decades [2, 3]. High capital cost and suitable reactor design (e.g., photo catalytic or heterogeneous catalytic) are required for practical applications of AOPs [4]. Combination of fluidized bed reactor with advanced oxidation processes (AOPs) has been recently studied by many researchers [5, 6]. Advanced oxidation processes completely mineralize recalcitrant compound and may produce nonhazardous by-products. Heterogeneous catalytic system has been found to be extremely efficient among all AOPs in degradation of complex chemical compounds and other industrial wastes [7]. The application of Fenton's reagent as an oxidant for wastewater treatment is a smart choice due to wide availability of iron, easier handling of nontoxic hydrogen peroxide, and efficient decomposition to environmentally safe products [8, 9]. There are many recent studies on the use of fluidized bed reactor for wastewater treatment using photo Fenton oxidation [10–12], heterogeneous Fenton oxidation [13, 14], ozone [15, 16], and homogeneous Fenton oxidation [17, 18]. In fluidized bed reactors, the solid particles fluidized by liquid or gas act as a fluid through the reactor. Compared to other types of reactors (e.g., fixed bed reactors), fluidized bed reactors have a number of advantages. Fluidized bed reactors can be considered as an improvement over the traditional water treatment methods associated with advanced oxidation processes for pollutant degradation. In this work an assessment was done by simulating FBR for treating wastewater using Fenton reaction.

Operation of FBR has confirmed many advantages that include high degradation efficiency, lesser reaction time and better catalyst recirculation [19]. However, there are some challenges with fluidized bed reactors. Fluidized beds are a heterogeneous mixture of fluid and solid particles; as a result proper description of the system is difficult to define. In FBR heterogeneous catalytic process, the oxidant (H_2_O_2_) is decomposed to highly reactive hydroxyl radicals in a catalytic sequence with ferrous ion as a catalyst [20]. Although FBR-AOPs have been studied much, few recent studies are available on modeling of FBR-AOPs and there is a lack of study in the prediction of performance of full scale FBR-AOP systems. Another major problem is scaling up a fluidized bed reactor and the limited availability of detailed studies that predict the performance of FBR-AOP systems.

Knowledge gap in modeling, scale-up strategies, and performance prediction is among the important factors hindering the expansion of commercial water treatments by FBR-AOP technology. Full-size commercial reactors are generally complicated and costly; so before coming into a final design of any industrial reactor, some validation of the data should be done. Mimicking hydrodynamics of lab-scale data can be a promising solution to this problem. But multiphase systems are complex in nature and scale-up of multiphase processes is highly unpredictable. Proper scale-up of any reactor requires hydrodynamic similarity in different scales. In addition validation of hydrodynamic properties at different scale is time consuming and costly by mean of experimental procedures. Under these circumstances, a theoretical method would be more appropriate to tackle the challenging problems for scale-up.

Therefore, a rule for designing a reactor process regardless of the involvement of any treatment or production can be proposed like this; firstly, to get well acquainted with the hydrodynamics, kinetics, heat transfer, and mass transfer involved in the system; secondly, to numerically solve the governing equations with help of simulation tools; and thirdly, to test scale-up methods available in the literature to come up with an approximate definition of the process. Phenol is used as an indicating contaminant for this work. Phenols and phenolic compounds are the most abundant pollutants in industrial wastewater due to their wide consumption in oil refineries, pulp and paper mills, resins, and steel coke manufacturing and pharmaceutical industries [8]. The average concentration of phenol found in wastewaters is 1.5 g/L, but the concentration can be as high as 4.5 g/L in highly polluted waters [21]. In the year 2002, the WHO (World Health Organization) fixed the limit of phenol in drinking water at 1 mg/L to avoid adverse health effects caused by phenols [22]. In this work 40–90 mg/L of phenol was used as input concentration for the proposed treatment system. However, treating phenol in wastewater to nontoxic level by a series of biological and chemical treatment processes is difficult due to quick solubility and steadiness of phenol in water [23]. Kinetics of phenol oxidation have been studied and reaction mechanisms have been narrowed down to 3 (three) consecutive equations for simulation input. However, catalyst ferrous sulphate and goethite have been used. However, Goethite (a-FeOOH) has recently been found to catalyze the oxidation of recalcitrant compounds by hydrogen peroxide [24].

Now, among other available simulation tools, computational fluid dynamic (CFD) simulations have been used in previous studies on AOPs for modeling chemical species transport. It is a tool that can simultaneously solve fluid dynamic equations in the course of space and time. There are some studies on use of CFD for catalytic reactors in water treatment [25, 26]. In addition, applying CFD analysis data to scale-up reactors minimizes experimental effort and fabrication costs at the pilot-scale level. The aim of this work is to develop a simulation model to describe the hydrodynamic and kinetic phenomena happening in the FBR. Total organic carbon (TOC) that represents surrogate information instead of specific species concentration was used to understand the removal efficiency of phenol in this study. Some of the previous reports on modeling have also been based on TOC [10, 27, 28].

Earlier results by some researchers have confirmed the significance of combining reactor hydrodynamics to predict the degradation process [29, 30]. However, there are few similar studies in phenol wastewater catalytic degradation in a fluidized bed reactor involving contribution of intermediate products. In the present study, the kinetics of TOC removal for initial 0.4 mM to 0.9 mM phenol concentration was investigated using goethite catalyst particles along with Fe^2+^ catalyst. A CFD model for simulation of catalytic fluidized bed reactor for water treatment was developed for the proposed fluidized bed reactor for better understanding. TOC concentration profile was determined by using CFD. This approach can help obtain a more specific understanding of design and optimization of fluidized bed reactor. Also, velocity profile was obtained in the developed fluidized bed reactor geometry with CFD simulation. The traditional Navier-Stokes equation was used to illustrate the flow through the liberated zone, while Brinkman equation was used to represent the flow through the fluidized zone. Some assumptions and boundary conditions have been considered along with the hydrodynamics, species mass transport, and chemical reaction kinetics in the modeled reactor. The simulated results show fluid velocity change and concentration profile of components with reactor height. Meanwhile TOC prediction by kinetic model was developed based on widely accepted reactions for phenol degradation in heterogeneous Fenton process. Finally, the computed result from CFD was used for scaling up the proposed bench-scale FBR to pilot-scale FBR. Similitude method was used for the scale-up procedure. Thus in this work phenol degradation by catalyst in a FBR process was modeled using computational fluid dynamic system and the results have been used with the help of similitude method for scale-up this 2-phase system.

## 2. Materials and Methods

**Figure 1:**
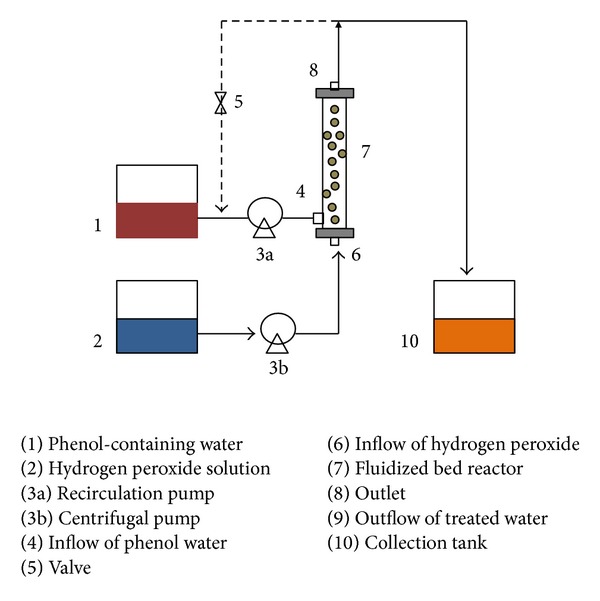
Schematic diagram of the proposed FBR-AOP system.

It has been revealed by some authors that batch recirculation systems are considerably preferred in degrading highly contaminated wastewater in terms of TOC reduction [31, 32]. In batch recirculation system, the contact between catalyst and pollutant is longer, which increases pollutant degradation efficiency. The schematic illustration of the proposed systems for phenol wastewater degradation is presented in Figure 1. The fluidized bed reactor consists of a tubular shape with two feed inlets and one outlet at the end of the reactor. Commercial grade goethite, FeOOH (Sigma Aldrich, Germany) was used in the catalytic bed and the carrier particles are glass beads of diameter of 1~2 mm. The voidage of the catalyst bed was calculated using the equation given by Antoni et al. as follows:

(1) ϵmf=0.586−0.7(η2ρfμdp3)0.029(ρfρs)0.021.

In the above equation,  *ϵ*_*mf*_ is the voidage of particles in minimum fluidization state, *η* is the viscosity of the fluid, *ρ*_*f*_ and *ρ*_*s*_ are the densities of the fluid and solid particle, *μ* is the dynamic viscosity of the fluid, and *d*_*p*_ is the diameter of particles.

The voidage was found to be 0.5989 for fluidized glass particles in water. The viscosity of the phenolic water was assumed to be the same as water. The velocity of fluidization for liquid-solid fluidization system was 0.03 m/sec, calculated using the formula given by Wen and Yu [33] as follows:

(2) $$\begin{array}{c} U_{mf}=7.90\times 10^{-3}d_{p}^{1.82}{\left(\rho_{s}-\rho_{f}\right)}^{0.94}\mu_{f}^{-0.88}, \end{array}$$

where (*ϕϵ*_*mf*_)^−1^ ≈ 14 and (1 − *ϵ*_*mf*_)/*ϕ* ^2^ *ϵ*_*mf*_^3^ ≈ 11.

In the above equation, *U*_*mf*_ is the minimum fluidization velocity, *d*_*p*_ is the diameter of particles, *ρ*_*f*_ and *ρ*_*s*_ are the densities of the fluid and carrier particle, *μ* is the viscosity of the fluid, and *ϕ* is the sphericity of the carrier particle.

The tube region packed with heterogeneous catalyst and glass beads in the amount of 32 g/L can occupy 70% of the reactor volume. Uniform distribution of catalyst was assumed in CFD simulation. 40–90 mg/L of phenolic water will be supplied into the system through one of the inlets, while hydrogen peroxide and water were pumped into the system through another inlet. The feed solution (water + phenol) was continuously recirculated by a pump. The fluidized bed reactor model is presented in Figure 1.  Table 1 represents the operating conditions for the reactor for two phenol concentrations and Table 2 shows the possible mechanism of reaction taking place in the catalyst fluidized bed, respectively. As for the degradation mechanism, phenol mainly gets oxidized to catechol and then benzoquinone before becoming water and carbon dioxide. The solution pH is to be maintained at 3 because high level of dissolved ferrous ions species is achieved in more acidic conditions [34].

CFD simulation described the TOC concentration profile inside the reactor module and predicted the TOC removal performance at different operating conditions for this process. The effects of pH change on TOC reduction were negligible [34, 40]. As it is assumed that the reactions took place only inside the catalyzed fluidized bed, the rise of concentration profiles in the bulk of liquid phase is insignificant.

## 3. Theory

### 3.1. CFD Model Development

Generally, application of fluidized bed in wastewater treatment technologies is complicated. In addition, there is a lack of knowledge in this area in terms of hydrodynamics and kinetics. In this study, we tried to define the hydrodynamics and kinetics in order to depict some important parameters, such as component concentration within the reactor length with the help of CFD simulation. Though several investigations have been conducted in AOPs modeling, there are very limited studies on CFD modeling for Fenton process [41]. CFD is a numerical technique that illustrates physical and chemical changes within the reactor by solving governing equations and their boundary conditions [30].

The catalyst bed of SiO_2_ and FeOOH was subjected to fluidize in the reactor. Only half of the reactor system was into consideration for simulation purposes as the reactor was symmetrical. The designed reactor consists of a cylindrical structure with two inflow tubes and one outlet (see Figure 1). Cylindrical coordinates were used for modeling the reactor. The incoming species and injected species reacted in the heterogeneous and homogeneous catalyst system in a completely fluidized state. The operating flow rate was based on previous design calculations. The reactor description and conditions are given in Table 1.

#### 3.1.1. Model for Kinetic Mechanism

This study concentrates on phenol degradation in the proposed system. Reaction kinetics of phenol degradation has been investigated in several previous studies [20, 39]. In general, complete oxidation of phenol to CO_2_ is not practically feasible due to high H_2_O_2_ consumption. As a consequence, it is necessary to study the concentration of toxic intermediates in the degradation mechanism. As this process is complex, various reaction schemes can be gathered to carefully analyze the mechanism. Following that, catechol, benzoquinone, and hydroquinone have been found as the main initial oxidation products that indicate hydroxylation taking place principally in the ortho ring position to the phenolic group [42]. Abundant products like maleic, acetic, and formic acids are produced when dearomatization process begins [43]. Rate reaction of oxalic acid is slower but attains higher concentration with time. Dicarboxylic, maleic, and muconic acids are important compounds as a result of ring opening of aromatic intermediates. The reactions involving phenol mineralization are shown in Table 2. The enormous number of reaction mechanisms for phenol degradation can be grouped based on production of aromatic compounds or acid and scavenging reactions.

The mechanism is reduced from reaction R1 to R18 based on widely accepted literature. R1 to R3 represent the formation of OH radical in reaction with Fe^3+^ from the catalyst and R4 to R6 represent scavenging effect of hydrogen peroxide. R7 to R12 represent production of aromatic compounds. R13 to R18 represent production of acids in degradation. As a whole, R7 to R18 represent production of intermediates. Complete mineralization regardless of production of intermediates is presented by R19 to R21. Reactions participation can be classified in three basic steps. First, the initiation and termination of radicals, hydrogen abstraction, and depropagation (decay/scission) [17, 44]. According to LCA (Long Chain Approximation), initiation and termination are neglected as they do not significantly affect the reaction rate [17, 45]. However, little is known about the sequence of intermediates formation. Following these findings, the total organic carbon, instead of phenol and intermediates, is taken as a surrogate parameter of organic matter present in water. TOC is assumed as the sum of the contribution of two types of compounds. The initial phenol concentration is expressed by TOC_1_ and the total formation of intermediate is expressed by TOC_2_. Based on the chain end scission in the population balance equation, the kinetic reactions in Table 2 can be summed up to three dynamic equations [2]:

(3) $$\begin{array}{c} {\text{H}}_{2}{\text{O}}_{2}⟶2{\text{HO}}^{∙} \end{array}$$

(4) $$\begin{array}{c} {\text{TOC}}_{1}+{\text{HO}}^{∙}⟶{\text{TOC}}_{2}+{\text{H}}_{2}\text{O} \end{array}$$

(5) $$\begin{array}{c} {\text{TOC}}_{2}+{\text{HO}}^{∙}⟶{\text{CO}}_{2}+{\text{H}}_{2}\text{O} \end{array}$$

These reactions are also supported by several authors [46]. This simplification is stated as general lumped kinetic model (GLKM) [8, 47, 48]. In this research, general lumped kinetic model is applied where the intermediate products (catechol, benzoquinone, hydroquinone, etc.) can be expressed as a set of first order ordinary differential equations. Concentrations of these various intermediates can be represented by a lumped concentration of total organic carbon.

Furthermore, the following reaction rate equations can be derived from (3), (4), and (5):

(6) $$\begin{array}{c} {\text{R}}_{{\text{HO}}^{∙}}={\text{k}}_{19}\left[{\text{H}}_{2}{\text{O}}_{2}\right] \end{array}$$

(7) $$\begin{array}{c} {\text{R}}_{{\text{TOC}}_{1}}={\text{k}}_{20}\left[{\text{HO}}^{∙}\right]\left[{\text{TOC}}_{1}\right] \end{array}$$

(8) $$\begin{array}{c} {\text{R}}_{{\text{TOC}}_{2}}={\text{k}}_{21}\left[{\text{HO}}^{∙}\right]\left[{\text{TOC}}_{2}\right] \end{array}$$

As our interest is on individual concentration profiles, rate expressions for reactant and products were determined. The rate expressions can be stated as follows:

(9) $$\begin{array}{c} r_{TOC_{1}}=-{\text{k}}_{20}\left[{\text{HO}}^{∙}\right]\left[{\text{TOC}}_{1}\right] \end{array}$$

(10) $$\begin{array}{c} r_{TOC_{2}}={\text{k}}_{20}\left[{\text{HO}}^{∙}\right]\left[{\text{TOC}}_{1}\right]-{\text{k}}_{21}\left[{\text{HO}}^{∙}\right]\left[{\text{TOC}}_{2}\right] \end{array}$$

(11) $$\begin{array}{c} r_{OH}=2{\text{k}}_{19}\left[{\text{H}}_{2}{\text{O}}_{2}\right]-{\text{k}}_{20}\left[{\text{HO}}^{∙}\right]\left[{\text{TOC}}_{1}\right]-{\text{k}}_{21}\left[{\text{HO}}^{∙}\right]\left[{\text{TOC}}_{2}\right] \end{array}$$

The three expressions stated above were selected for the simulation purpose based on the kinetic information from the literature.

#### 3.1.2. Reactor Hydrodynamics

In the proposed reactor liquid (phenol water) and solid interaction (catalyst and glass beads) will result into effective degradation of phenol. Numerical simulation of liquid flow and transport in a fluidized bed reactor is challenging as the liquid flows continuously, but the catalyst bed creates a porous zone for the liquid. On this note, this liquid movement through the reactor can be described by two steps. Firstly, free-flow of the liquid through the system and, secondly, fluid flowing through fluidized-state porous system. The momentum balance for the first step can be described with incompressible Navier-Stokes equation. The stationary Navier-stokes equations describing the fluid flow in the liberal flow regions is as follows:

(12) ∇·[−μ((∂2uz∂x2+∂2uz∂y2+∂2uz∂z2)+ (∂2uz∂x2+∂2uz∂y2+∂2uz∂z2)T)+ pI]  =−ρ(u·∇)u,

where, *ρ*, **u**,  *p*, *μ*, ∇, and *T* are density, velocity vector, pressure, dynamic viscosity, del operator, and deviatoric component of the total stress tensor, respectively. The force vector “**F**” is omitted for the absence of any pneumatic probes in the reactor premises.

In the second step, the flow through the porous fluidized catalyst bed is described by a combination of the continuity equation and momentum balance equation, which is Brinkman equation [37, 38, 44–50]. Radial, axial, and tangential velocities and pressure are the dependent variables in Brinkman equation. The flow in porous media is governed by the following expression:

(13) ρεp((u·∇)uεp) =∇·[−pI+μεp(∇u+(∇u)T)−2μ3εp(∇·u)I]  −(μκbr+βF|u|+Qbr)u+F.

*μ* denotes the dynamic viscosity of the fluid (SI unit: kg/(m*·*s)), **u** is the velocity vector (m/s), *ρ* is the density of the fluid (kg/m^3^), *p* is the pressure (Pa), *ε*_*p*_ is the porosity, *κ*_br_ is the permeability of the porous medium (m^2^), ∇ is the del operator, *T* is the deviatoric component of the total stress tensor, respectively, and *Q* is a mass source or mass sink (kg/(m^3^ *·*s)). Influence of gravity and other volume forces can be accounted for using the force term **F** (kg/(m^2^ *·*s^2^)).

Brinkman equation in *x*-direction is

(14) ρεp(∂ux∂t+ux∂ux∂x+uy∂uy∂y+uz∂uz∂z) =[−∂p∂x+μεp((∂2ux∂x2+∂2uy∂y2+∂2uz∂z2)+(∂2ux∂x2+∂2uy∂y2+∂2uz∂z2)T)]  −2μ3εp(∂2ux∂x2+∂2uy∂y2+∂2uz∂z2)I  −(μκbr+βF|u|+Qbr)u+Fx.

Brinkman equation in *y*-direction is

(15) ρεp(∂uy∂t+ux∂uy∂x+uy∂uy∂y+uz∂uy∂z) =[−∂p∂x+μεp((∂2uy∂x2+∂2uy∂y2+∂2uy∂z2)+ (∂2uy∂x2+∂2uy∂y2+∂2uy∂z2)T)]  −2μ3εp(∂2uy∂x2+∂2uy∂y2+∂2uy∂z2)I  −(μκbr+βF|u|+Qbr)u+Fy.

Brinkman equation in *z*-direction is

(16) ρεp(∂uz∂t+ux∂uz∂x+uy∂uz∂y+uz∂uz∂z) =[−∂p∂x+μεp((∂2uz∂x2+∂2uz∂y2+∂2uz∂z2)+(∂2uz∂x2+∂2uz∂y2+∂2uz∂z2)T)]  −2μ3εp(∂2uz∂x2+∂2uz∂y2+∂2uz∂z2)I  −(μκbr+βF|u|+Qbr)u+Fz.

The term (**u** · ∇)**u**/*ε*_*p*_ can be disabled as Reynolds number is much higher than 1. The Forchheimer drag option adds a viscous force proportional to the square of the fluid velocity **F**_**F**_ = −*β*_**F**_|**u**|**u**. The Forchheimer term *B*_**F**_ has units of kg/m^4^. The mass source, *Q*, accounts for mass deposit and mass creation in domains, and the mass exchange is assumed to occur at zero velocity.

The dependent variables are the concentrations of the reactants and products. Inertial term for Stokes flow and porous media are taken into account in this case.

The equation can be presented as follows after simplification:

(17) $$\begin{array}{c} \nabla \cdot \left[-\frac{\mu }{\varepsilon_{p}}\left(\nabla u+{\left(\nabla u\right)}^{T}\right)+pI\right]=-\frac{\mu }{k}u. \end{array}$$

The equation in *x*-direction is

(18) ∇[−μεp((∂2ux∂x2+∂2uy∂y2+∂2uz∂z2)+(∂2ux∂x2+∂2uy∂y2+∂2uz∂z2)T)+pI]=−μκu.

The equation in *y*-direction is

(19) ∇[−μεp((∂2uy∂x2+∂2uy∂y2+∂2uy∂z2)+(∂2uy∂x2+∂2uy∂y2+∂2uy∂z2)T)+pI]=−μku.

The equation in *z*-direction can be stated as

(20) ∇[−μεp((∂2uz∂x2+∂2uz∂y2+∂2uz∂z2)+(∂2uz∂x2+∂2uz∂y2+∂2uz∂z2)T)+pI]=−μku.

#### 3.1.3. Species Mass Balance

Continuity equation was used for each compound for describing mass balance in this fluidized bed reactor. Simultaneous solving of the equation is the key step to deduce the concentration profile of TOC inside the fluidized bed reactor. It was assumed in the system that the modeled species were in very low concentrations compared to solvent liquid. Fickian approach was used for the diffusion term in mass transport. The model of 19 species and 16 reactions was done based on convection and diffusion equation. The equation is as follows:

(21) ∂∂r(−Di(∂ci∂x+∂ci∂y+∂ci∂z)−ziumFci(∂Vi∂x+∂Vi∂y+∂Vi∂z))  +u·(∂ci∂x+∂ci∂y+∂ci∂z)=Ri.

In the above equation, *D*_*i*_ is the diffusion coefficient of species *i* (m^2^/s), *C* is the concentration of species *i* (mol/m^3^), **u** is the fluid velocity (m/s), **F** refers to Faraday's constant (A*·*s/mol), *V* denotes the electrical potential (*V*), *z*_*i*_ is the charge number of the ionic species (unit less), **u**_*m*,*i*_ is the ionic mobility, and *R* is the production or consumption rates expression for the species (mol/(m^3^ *·*s)). This equation can be simplified to the following expression:

(22) $$\begin{array}{c} \frac{\partial }{\partial r}\left(D_{i}\left(\frac{\partial c_{i}}{\partial x}+\frac{\partial c_{i}}{\partial y}+\frac{\partial c_{i}}{\partial z}\right)+C_{i}u\right)=R_{i}. \end{array}$$

In this equation, *c*_*i*_ denotes the concentration (mol/m^3^), *D*_*i*_ the diffusivity (m^2^/s), and *R*_*i*_ the reaction rate for species *i* (mol/m^3^ *·*s).

This mass balance equation occurs for both diffusion and convection in a conservative manner. It means that the terms from the conventional part *c*∇·**u** become zero for an incompressible fluid. This ensures that nonphysical source terms cannot come from the solution of a flow field.

The expression term presented in equation *A* is called flux node and it defines the total flux of species *c*. This is used to specify the total species flux across the boundary:

(23) Ni=(−Di(∂ci∂x+∂ci∂y+∂ci∂z)− ziumFci(∂Vi∂x+∂Vi∂y+∂Vi∂z))+Ciu.

At the outlet, convection is assumed to dominate the mass transport:

(24) $$\begin{array}{c} n\cdot \left(-D_{i}\left(\frac{\partial c_{i}}{\partial x}+\frac{\partial c_{i}}{\partial y}+\frac{\partial c_{i}}{\partial z}\right)+c_{i}u\right)=n\cdot c_{i}u. \end{array}$$

A common assumption for tubular reactors is that the gradient of *c*_*i*_ in the trend perpendicular to the outlet boundary is insignificant; (24) indicates the same. There is a high degree of transport by convection in the direction of the main reactor axis. This stipulation eliminates the requirement for stating a fixed concentration value for the flux at the outer boundary. Thus, insulating conditions apply at all other boundaries and the equation is as follows:

(25) $$\begin{array}{c} n\cdot \left(-D_{i}\left(\frac{\partial c_{i}}{\partial x}+\frac{\partial c_{i}}{\partial y}+\frac{\partial c_{i}}{\partial z}\right)+c_{i}u\right)=0. \end{array}$$

The disclosed governing equations (see (12) to (25)) can explain physical and chemical changes within a reactor mutually [30].

*Applied Assumptions and Boundary Conditions.* The simulation of the reactor depends on the implicated boundary conditions and model volume. The assumptions for the simplification of the process model are presented below.

**Figure 2:**
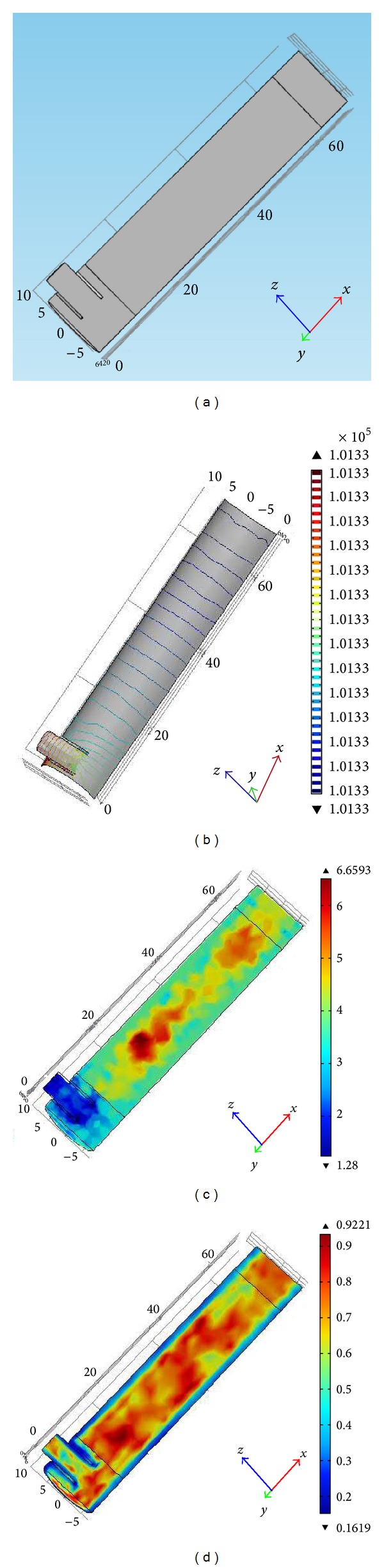
(a) Schematic diagram of the reactor (dimensions are in centimeters), (b) pressure change across the reactor, (c) mesh quality size, and (d) mesh element size.

- Phenol is degraded by reaction with hydroxyl radical.
- Unsteady reactions and states are considered in the system for better understanding.
- Isothermal condition is assumed in the reactor system, as Fenton oxidation itself is not heat consuming and there is no significant difference found between the reactor's inlet and outlet temperature. Furthermore, isothermal assumption reduces computational demand in the simulation.
- The fluid flow is incompressible, as the flow is defined as laminar and the fluid flow is Newtonian.
- The substantial properties of the mixture (e.g., viscosity, density, etc.) are assumed to be independent of the mass fraction of the components.
- The porosity and void age of the fluidized bed reactor are considered to be in constant state in the simulation system.
- It is assumed that the reaction only takes place in the fluidized region so the reaction rate term is zero in free-flow region.
- The catalyst bed of this fluidized bed reactor is assumed to be fully developed and the reacting zone is from 10 cm to 65 cm giving a height of 55 cm of reacting zone (see Figure 2).
- Time-dependent solution is achieved considering an appropriate time step.
- Due to isothermal condition, energy balance is not employed.

Boundary conditions for the catalytic fluidized bed reactor length are given as follows.

- The boundary condition for the inlet is the known value of the velocity, no slip condition for the walls of the reactor.
- It is assumed that the fluid is Newtonian, incompressible, isothermal, and nonreactive with constant physical properties and under laminar steady state flow. The hydrodynamic and species transport gives mass conservation equation: ∇·**u** = 0.
- A constant velocity profile is assumed at the inlet boundaries; **u** = **u**_in_.
- The boundary condition for the Navier-Stokes equations at the outlet reads *t* · **u** = 0, *p* = 0, where *t* is any tangential vector to the boundary.
- At the inlet, **u** = 0.03 m/s, **u** is calculated by (1).
- At the inlet, gradients of **u**_*x*_, **u**_*y*_, and **u**_*z*_ are set to zero.
- At outlet, pressure is set to standard atmospheric pressure at 101,325 Pa (see Figure 2).
- No flux condition is applied for exterior wall which is represented by the following equation-*n* · *N*_*i*_ = 0.
- The concentration of the inlet is fixed: *c*_*i*_ = *c*_*i*0,inlet_ for the mass transport.
- At the inlet, *c* is set to zero.

*Reactor Geometry and Meshing*. The geometry of the reactor was defined after determining the governing equations and boundary conditions. The fluidized bed reactor of 70 cm-height and 7 cm-diameter was established on COMSOL multiphysics (Version 4.2.a), available in Department of Chemical Engineering, University of Malaya. Reactor geometry is shown in Figure 2 and the units are in cm. This reactor geometry was solved in both time dependent solver and stationary solver. CFD simulation and modelling enable performance prediction of a real system and meshing of the element improves the accuracy of the simulation results. Therefore, hexagonal meshing was done to the geometry for accurate prediction in this study. The simulated reactor was of two-phase flow in laminar regime and the bed reactor with the fluid flow was descriticized in small elements. The mesh quality is shown in Figure 2.

A set of 3D models using finite element method (FEM) were considered for time dependent simulation of the mentioned system. The transport equations were described by Navier-Stoke for fluid flow, Brinkman equations for porous media, and Stephan-Maxwell equations for conversion rate of reaction and convection diffusion mechanisms. Besides, the parallel sparse direct linear solver (PARDISO) algorithm was applied to combine and solve the equations. This algorithm is a direct sparse solver which supports parallel processing. Equations (12) to (25) were solved numerically using COMSOL multiphysics (Version 4.2.a). The finite volume methods (FVM) were used to solve the governing equations over discrete control volume. Additionally, PARDISO was used [50]. The kinetic parameters were estimated by trial and error.

### 3.2. Scale-Up Strategy

Scaling up a reactor system is possible when there are available data of velocity or pressure drop or Reynolds number, and so forth, in different positions of the system. From CFD data of velocity, information has been gathered with change in reactor height of the proposed system. For successful scale-up of a system, a number of scale independent factors are required. However, when reactions are involved in the system it becomes quite difficult to maintain similar scale independent factors as mass transfer is highly scale dependent. The basic purpose is to obtain a set of* independent* dimensionless groups of the FBR system. For this reason, Buckingham's *π* theorem has been chosen to derive the dimensionless parameters from the operation and physical properties [51]. Buckingham's *π* theorem states that, if two beds are designed and operated to have identical values of all independent nondimensional parameters, then the dependent variables of the two beds must also be identical at every location within the bed, and* hydrodynamic similarity* is said to be achieved [52].

Again, to study the scale-up of this FBR similitude method developed by Glicksman and his coworkers was used. Although this method was developed for the scale-up of* gas-solid* fluidized beds, including bubbling beds and circulating fluidized beds [3, 53, 54], the same methodology can be extended to the* liquid-solid fluidization*, because of the similar governing mechanisms in fluid-solid contact, reactor construction, and operation. In this work, effort is specifically made to apply the similar scale-up methodology to liquid-solid fluidized bed by the similitude method [51]. Similitude method or dimensional analysis has been shown as a powerful tool to help scale-up between a full-size prototype and a laboratory-scale model, particularly in situations where the equations governing a physical problem are either unknown or not easily solved. Few things must be considered for scaling up a solid-liquid fluidized bed reactor; the hydrodynamic system should be governed by some dimensionless parameters, which can be kept similar throughout the scaling up process. Few design parameters are scale independent such as minimum fluidization velocity and bed density [55]. The simplified set of dimensionless parameters to be held constant for hydrodynamic similarity are as follows: *U*_*O*_^2^/*gD*, *ρ*_**F**_/*ρ*_*S*_, *U*_*O*_/*D*, *d*_*p*_/*D*, *ρ*_*s*_ *ρ*_*f*_ *d*_*p*_^3^ *g*/*μ*_*f*_^2^ and dimensionless particle size distribution. Five types of scale reactors (RCTR1, RCTR2, RCTR3, RCTR4, and RCTR5) were chosen to be analyzed for scale-up. The physical properties of these reactors are given in Table 4. And in this study similar dimensionless numbers were achieved with diameter change of *m* times larger reactor with the following relation for diameter of the reactor:

(26) $$\begin{array}{c} D_{m}=mD_{\left(m-0.5\right)}. \end{array}$$

Here *m* refers to *m* time's larger reactor in case of diameter and velocity. The minimum fluidization velocity *U*_*m*_ and the gas superficial velocity *U*_**F**_ were obtained by the relation suggested by Horio et al. [56], which is as follows:

(27) $$\begin{array}{c} \sqrt{m}=\frac{U_{mf}^{l}}{U_{mf}^{s}}=\frac{{\left(U_{F}-U_{mf}\right)}^{l}}{{\left(U_{F}-U_{mf}\right)}^{s}}, \end{array}$$

where the superscript *s* refers to the smaller bench-scale model and the superscript *l* refers to the *m times* larger scale model. Horio et al. [56] generated correlation for conversion, selectivity, and yield using two-dimensionless parameters, which are mass transfer number, *N*_*m*_, and reaction number, *N*_*r*_. These two parameters will change with scale change *Z*. Here, *Z* is the dimensionless distance along the reactor height. *N*_*m*_ can be considered to follow the same equation for all reaction orders:

(28) $$\begin{array}{c} N_{m}=\frac{k_{\text{bea}}H}{U_{L}}. \end{array}$$

Here, *k*_bea_ is a coefficient which will vary with scale change in the reactor. The interchange coefficient was gas interchange coefficient in the work of Horio et al. [56]. But in our work there is no presence of gas. In this case, another coefficient can be taken into account which will change according to reactor scale change, and this is the particle velocity. Solid interchange coefficient will directly affect our phenol degradation. Therefore, the same interchange equation has been used replacing bubble diameter with particle diameter and minimum fluidization with fluid velocity. According to the correlation by Davidson and Harrison 1964, [57, 58] the interchange coefficient is given by

(29) $$\begin{array}{c} k_{\text{bea}}=4.5\frac{U_{T}\varepsilon_{p}}{d_{p}}+5.85\frac{D^{1/2}{\varepsilon_{p}g}^{1/4}}{d_{p}^{5/4}}. \end{array}$$

The first term in the above correlation represents the convection term and the second represents the contribution due to solid interchange. In this work convection term is changing, but the diffusion term is constant. For deriving reaction number, *N*_*r*_ the reaction mechanism of the system must be known. The simulation of our system was done considering (3), (4), and (5). The desired product of this system is mentioned as CO_2_ and H_2_O, which is meaning complete mineralization of the compound. The yield of any process significantly depends on the rate limiting reaction of the system. From our observations in the simulation study, it can be said that reaction (4) and reaction (5) are the principle reactions. Thus in this scale-up work those following two reactions are of concern:

(30) TOC1+HO∙→k20TOC2+H2O:RTOC1  =k20[HO∙][TOC1]

(31) TOC2+HO∙→k21CO2+H2O:RTOC2  =k21[HO∙][TOC2]

Here, the reactants TOC_1_ and HO^∙^ react on the surface of the catalyst to form the desired product, *P*. First, TOC_1_ and HO^∙^ react to produce intermediate TOC_2_ *·*HO^∙^ that further reacts with TOC_2_ to form the product *P*. All reactions are considered isothermal. Because of having two parallel equations involved, two values of reaction number *N*_*r*1_ and *N*_*r*2_ should be calculated. Both the reactions (30) and (31) are second order reaction. And for second (2nd) order reaction, *N*_*r*_ can be calculated by the following equation:

(32) $$\begin{array}{c} N_{r1/r2}=3600\frac{{\text{k}}_{20/21}\cdot H}{{U_{L}C}_{\text{phenol}}}. \end{array}$$

Reaction number *N*_*r*1/*r*2_ is seen to be changing with scale-up whereas the mass transfer number *N*_*m*_ stays the same. For this reason it is expected to have reactant conversion factor,  *x*_*A*_ yield,  *y*_*A*_ and selectivity, and *s*_*p*_ to be changing with scale change, *H* [52]. The conversion, yield, and selectivity for two parallel reaction mechanisms are given as below:

(33) xA=1−exp⁡(−NmNr1Nm+Nr1),yA=(Nr1Nr1−Nr2)[exp⁡(−NmNr2Nm+Nr2)−exp⁡(−NmNr1Nm+Nr1)],sp=(Nr1Nr1−Nr2)×((exp⁡(Nm2(Nr1−Nr2)(Nm+Nr1)(Nm+Nr2))−1)  × (exp⁡(NmNr1(Nm+Nr1))−1)−1).

These equations are functions of only the dimensionless reaction and mass-transfer numbers appearing in the model. For achieving similarity in a particular performance index during scale-up, the analytical expressions for the performance index for the small-scale, as well as the large-scale, reactor are equated. Then, the appropriate scale-up criteria can be identified by taking into account the variation of various dimensionless parameters with the reactor scale.

## 4. Results and Discussion

### 4.1. CFD Study

The CFD simulations of the fluidized bed reactor were established with COMSOL multiphysics. The free flow velocity profile was achieved by simultaneous solution of continuity and momentum equations along with their respective boundary conditions. Hydrodynamic results were obtained and reaction kinetics was implemented in a developed hydrodynamic condition.

**Figure 3:**
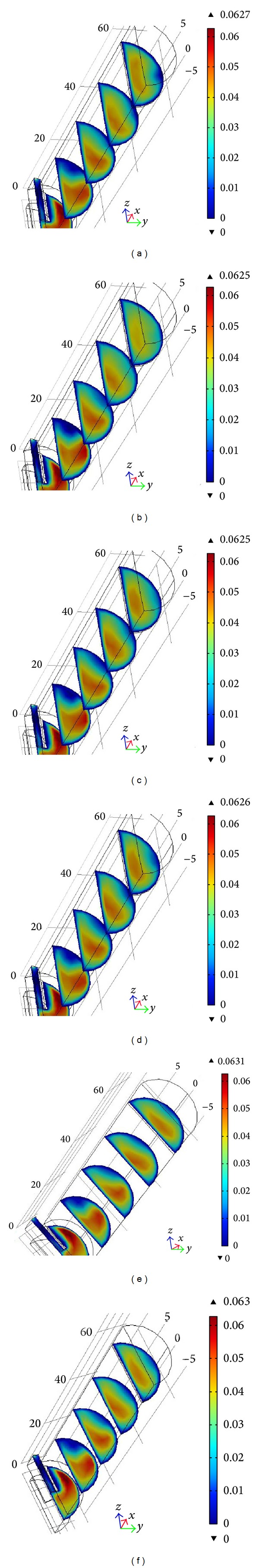
CFD simulation of velocity at (a) time = 150 seconds, (b) time = 350 seconds, (c) time = 550 seconds, (d) time = 750 seconds, (e) time = 1000 seconds, and (f) time = 2000 seconds.

**Figure 4:**
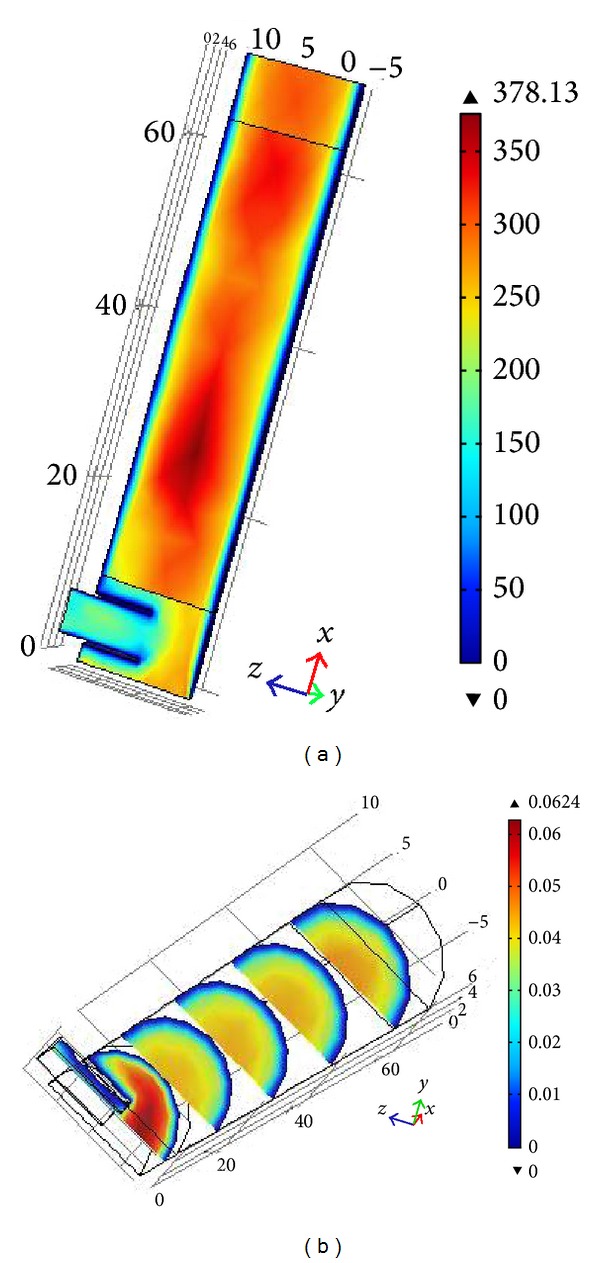
CFD simulation of (a) Reynolds number stationary-state volume plot and (b) slice plot for velocity in stationary state.

The 3D-model of the fluidized bed reactor was first solved for a time dependent laminar flow in absence of any reaction.  Figure 3 shows the slice plot for velocity magnitudes at different times. Velocity of higher magnitudes was observed at the inlet of the fluidized bed reactor for the free flowing zone. Lower velocity was attained in the centre of the fluidized catalytic bed due to the porosity of the fluidized catalysts and carrier. The velocities near the walls indicated that there was no slip condition in the reactor walls. There was no force vector applied on the velocity as well and the flow of fluid was smooth and continuous, ensuring complete fluidization. In order to determine the fully developed laminar velocity profile in the reactor, a 1D profile was generated portraying velocity development from the starting point of the reactor to the end of the fluidized state (see Figure 3). The Reynolds number achieved in the reactor was around *Re* = 300 which confirmed that the flow was laminar. A volume plot at stationary state of Reynolds number was presented in Figure 4.

No external velocity restrictions or internal force field were positioned on the outlet, inlet, and the gradients of all variables, except for pressure, which was set to zero in the flow direction at the outlet. The inlet velocity ranged from 0.03 to 0.05 ms^−1^, which corresponded to a flow rate of 3–5 L/min. All inlet velocities tangential to the inlet plane were set to zero. It can be seen from the 3D velocity progression along the reactor axis that the flow was moderately homogeneous in the catalytic area governed by Brinkman equation (see Figure 2). The indiscriminate arrangement of the glass particles support allowed a moderately good distribution of water flow and subsequently a uniform flow field through the reactor.

**Figure 5:**
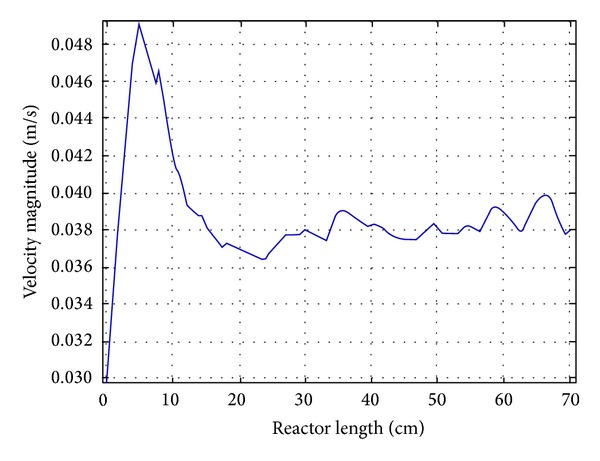
A one-dimensional view for velocity profile through the reactor.

**Figure 6:**
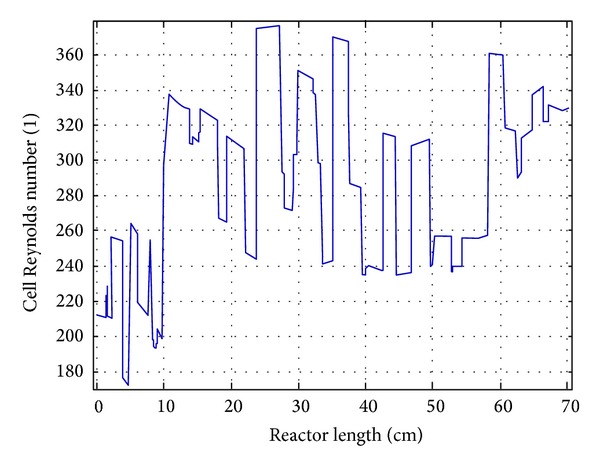
A one-dimensional view for Reynolds number through the reactor.

A 1D line was cut from the 3D reactor volume at the middle point in the reactor system for better understanding of normal axial velocity distribution (see Figure 5). This figure shows the inlet and outlet velocity of the reactor. It is clear from the graph that the fluid started to flow through the fluidized state at 20 cm of the reactor length and a slight drop of velocity could be seen from that point. The velocity through the fluidized state was almost uniform which confirmed uniform void age and porosity through the fluidized state. The average Reynolds number experienced was approximately *Re* = 300 throughout the reactor (see Figure 6).

### 4.2. Kinetic Study

**Figure 7:**
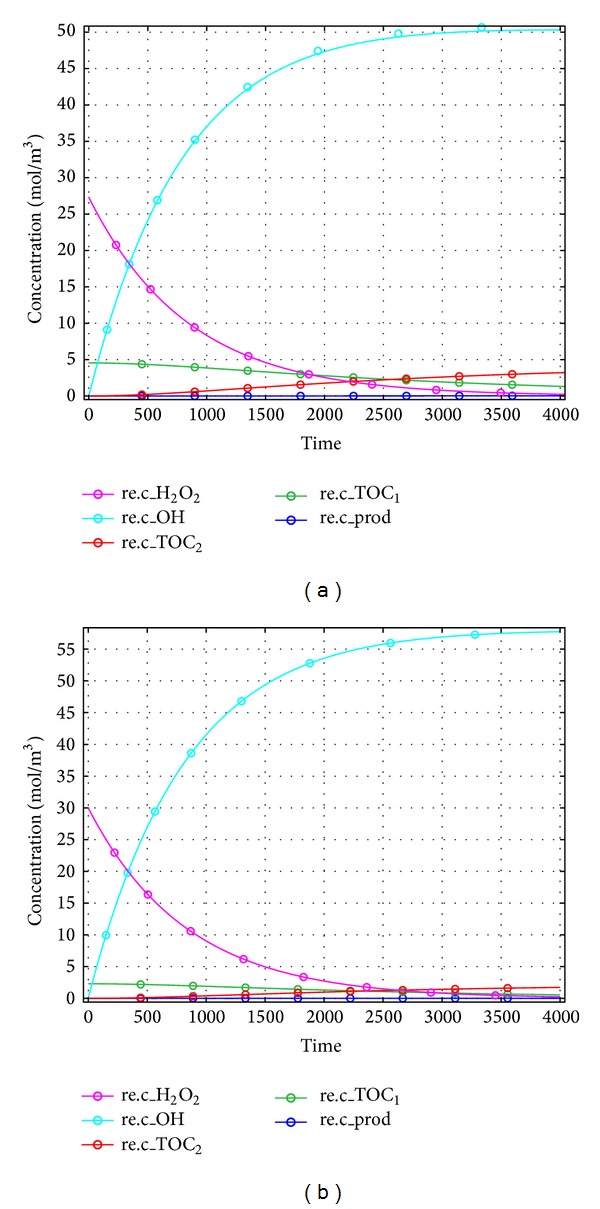
A one-dimensional view for concentration profile through the reactor.

**Figure 8:**
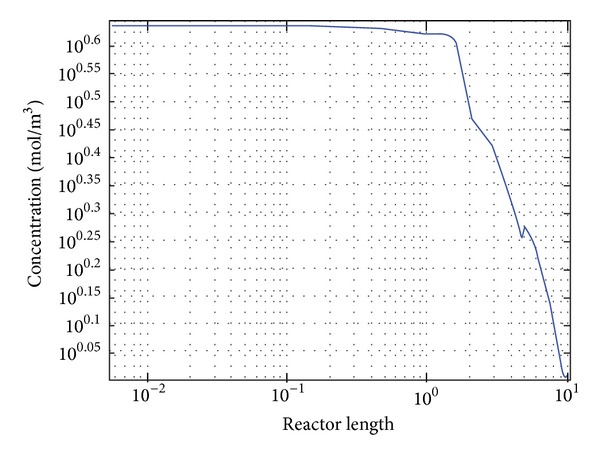
A one-dimensional plot for CFD simulation for TOC along with axial axis in mol/m^3^(integrated value).

Our aim is to degrade phenol in the fluidized state of catalyst and solution inside the reactor. For this purpose, the hydrodynamics of the reactor was simulated in absence of any reaction, and then the reactions were introduced to the system. There are 21 reactions involved in phenol degradation (see Table 2). These widely accepted reactions were simplified based on TOC to three consecutive reactions (R19 to R21). The kinetic rate constants of the reactions were found by trial and error. The originated kinetic rate constants (k_19_ to k_21_) were used to produce a 1D graph to depict the degradation and production of intermediate compounds in the reactor system over time. Figures 7(a) and 7(b) show the progress of phenol degradation by Fenton reaction for a reaction time of 4000 s.  Figure 8 represents concentration decrease with reactor length.

**Figure 9:**
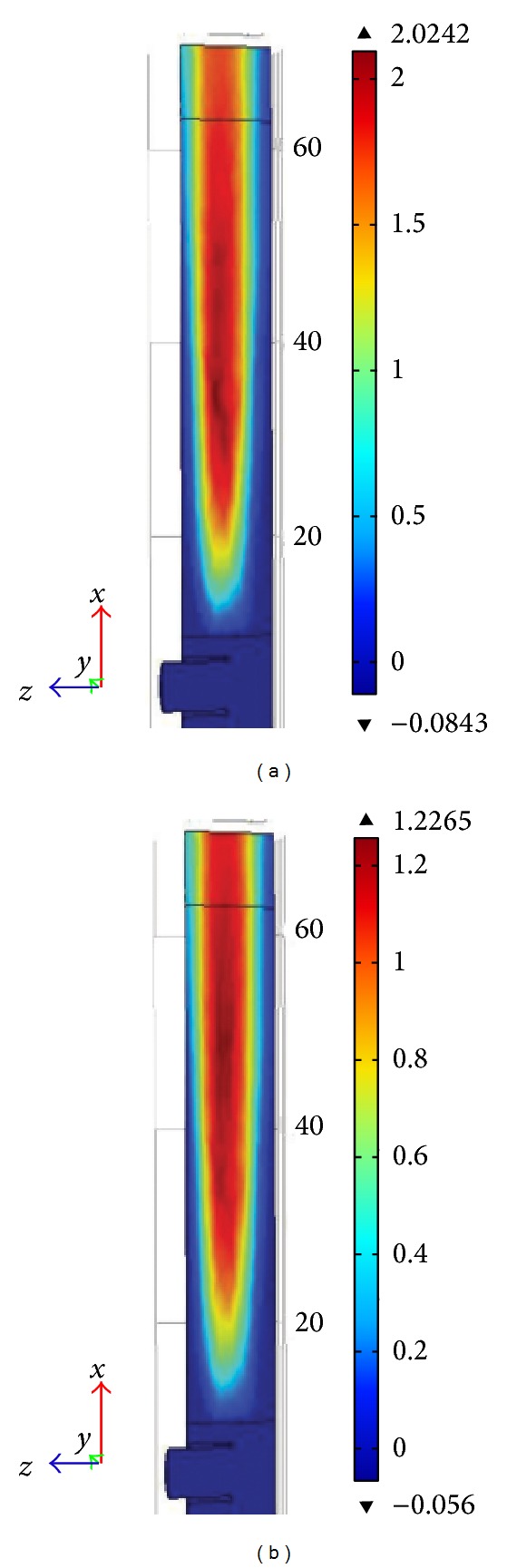
3D plot for CFD simulation for TOC_2_ (intermediate formation) concentration profile in mol/m^3^ (a) for initial TOC of 4.46 mol/m^3^ and (b) for initial TOC of 2.55 mol/m^3^.

Concentration profiles of phenol (TOC_1_), intermediates (TOC_2_), and hydroxyl radicals (OH) were obtained by solving governing mass transfer equations (see (21) to (25)) with solved module for kinetic equations. The concentration profile is presented in Figure 9. As illustrated in this figure the TOC of intermediates was increasing. Higher TOC generation due to production of intermediates can be seen starting from the catalyst bed of the reactor. Longer residence time of reacting solution inside the reactor resulted in higher TOC generation by intermediates and thus higher phenol removal. It can be understood from the figures that higher residence time is needed to complete the reaction.

In these reactions, the increase in intermediate concentration was followed by a decrease in phenol concentration. This was followed by an increase in hydroxyl radical production. The life cycle of hydrogen peroxide was around 3000 sec. The CFD results gave two second order reaction rates for intermediate formation and end product conversion. It was apparent from our findings that the conversion of the reaction increased with reaction time until the equilibrium point, which reached the maximum of 44 to 48%. But in the simulated reactor, the conversion was 48% and 46%, respectively; which had 9% and 4.16% deviations (see Table 3). The graphs show the molar concentration of TOC at steady state condition shown in Figure 9. Based on the 3D CFD modeling of the Fenton like degradation of phenol, ratio of kinetic rate constants for production of hydroxyl radicals (k_19_), production of intermediates (k_20_), and production of desired end products (k_21_) were on good agreement with previously published results. The kinetic rate constants for reactions (3), (4), and (5) were found to be 0.0012 s^−1^, 6.35 × 10^−5^ M^−1^s^−1^, and 9.6 × 10^−7^ M^−1^s^−1^, respectively. These kinetic constants were achieved by trial and error on the simulated system to get TOC removal % closer to the experimental TOC removal percentage.

### 4.3. Scale-Up Study

**Figure 10:**
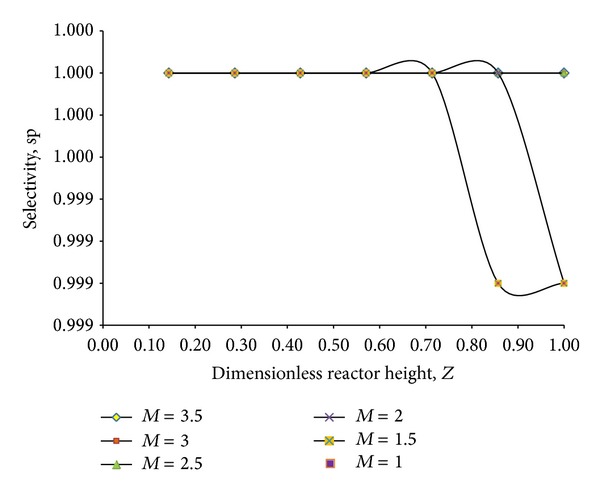
Screening of the reactors based on their hydrodynamic similarity with simulated reactor.

Five reactors were examined for scale-up from the simulated reactor. These five reactors were *m* (e.g., *m* = 1.5, *m* = 2, *m* = 2.5, *m* = 3, and *m* = 3.5) times larger than the bench scale reactor. Minimum fluidization velocity for each reactor (RCTR1, RCTR2, RCTR3, RCTR4, and RCTR5) was calculated according to (27).  Table 5 represents seven chosen dimensionless numbers for all the reactors. Theoretically if these dimensionless numbers can be kept the same for all of them, it can be expected that the hydrodynamic description will be the same for all. Although density ratio and velocity to diameter ratios were similar, change in other dimensionless numbers is seen with increasing scale-up of the bench-scale reactor. From this, some solutions can be depicted for achieving hydrodynamic similarity. For example, it can be said that particle diameter is a factor which was not changed and thus nonsimilarity resulted. Also, the similarity on dimensionless numbers can be achieved with increasing viscosity of the liquid; otherwise the fluid may not follow laminar attitude throughout the scaled up reactor unlike the simulated FBR. It has been observed that reactor diameter had no potential effect when the reactor diameter was increased. It is expected that making these scaling dimensionless groups equal would ensure the hydrodynamic similarity between two reactors. To monitor and screen the scaled up reactors, the hydrodynamic similarity must be looked at. Selectivity versus dimensionless reactor height for these reactors is presented in Figure 10.

**Figure 11:**
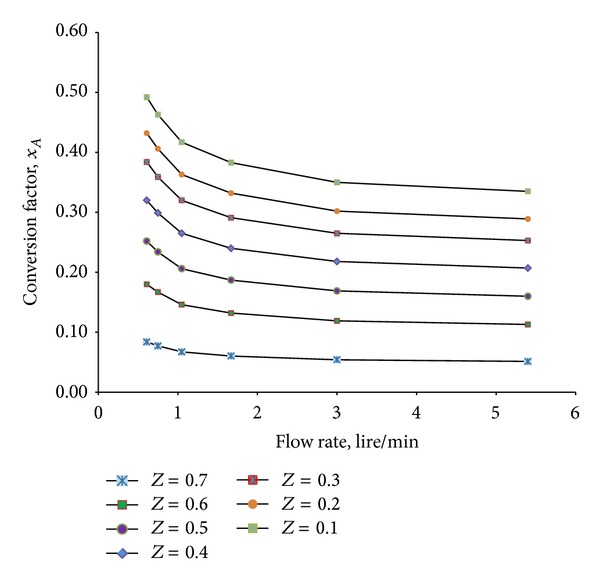
Comparison of conversion factor at different reactor lengths with changing flow rate.

In the above, Figure 10, we can see that *m* = 1 and *m* = 1.5 have similar hydrodynamics and *m* = 2 follows hydrodynamic similarity as *m* = 1 till dimensionless reactor length of 0.2. After 0.2 the hydrodynamic is no longer similar. Again for *m* = 2.5, *m* = 3, and *m* = 3.5 the hydrodynamic is completely different from *m* = 1 reactor. Similarity in hydrodynamics for these reactors can only be achieved with increased particle diameter and viscosity which will not be possible for the proposed reaction criteria of phenol degradation. Also with increasing reactor geometry the flow rate of solution is changing and so is the conversion factor.  Figure 11 represents the change in conversion factor through reactor height with increase in flow rate. For the simulated reactor the conversion of TOC_1_ is calculated to be 0.48 at the height of the reactor and the same factor is found for RCTR1 with *m* = 1.5. But with increasing flow rate the factor is shifting away.

The scaling up process of the simulated reactor revealed that reactor diameter has no significant effect on the reactor performance for phenol degradation. Actually, no change in hydrodynamics was observed with change in reactor diameter, given with constant reactor height. The hydrodynamic behaviour did not change with increasing or decreasing the reactor diameter for the observed numbers of m. Conversely, reactor performance was increased with lowering of initial phenol or TOC concentration. It can be explained by the fact that the reaction is of second order and the rate constant is distinctively slower; thus reactor performance could be enhanced only by decreasing the pollutant input. And also, generally systems dominated by reactions do not need strict maintenance of a bed diameter.

**Figure 12:**
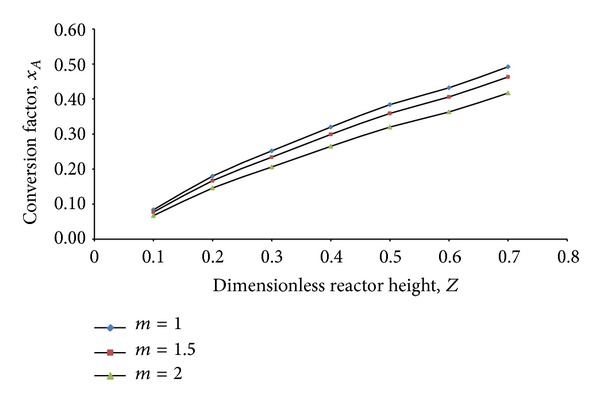
Reactor performance of similar hydrodynamic reactors (*m* = 1, *m* = 2, and *m* = 3) based on conversion factor versus dimensionless reactor  length.

For having almost similar hydrodynamics as the simulated reactor (*m* = 1), RCTR1 (*m* = 1.5) and RCTR2 (*m* = 2) are expected to give similar reaction mechanism as the proposed simulated reactor.  Figure 12 shows the conversion factor achieved through reactor length for *m* = 1, *m* = 2, and *m* = 3 reactors. It can be described from the illustration that the conversion factor slightly shifts downward with increase in reactor size. An alternate solution is to decrease the input phenol/TOC concentration to achieve similar effectiveness. This solution is drawn as there will be no improvement in performance with further geometrical change. Rather it will drop down to unacceptable limit. Properties change cannot be a solution as these properties (density, viscosity, and particle size) are fixed for treatment processes. Therefore, it can be concluded that RCTR1 can be a promising scaled up version of the simulated reactor, which can attain similar reactor performance, following similar hydrodynamics.

## 5. Conclusion

Treating recalcitrant aromatic compounds by various conventional treatment methods is challenging. Phenol water is hazardous to our environment; thus phenol has been chosen as a model contaminate for this work. A fluidized bed reactor process is proposed here for Fenton degradation of phenol water (40–90 mg/L). A hydrodynamic study was performed to describe phenol degradation in a 2.8 L volume catalytic fluidized bed reactor. The velocity change data in this simulation was used to produce scaled up values (using (26) and (27)) for five reactor geometries at dimensionless reactor length of (*Z* = 0.1, 0.2, 0.3, 0.4, 0.5, 0.6, and 0.7). Hereafter, similitude method was used to assess hydrodynamic and performance similarity between the reactors. The conclusions of this work can be stated as below.

1. Brinkman equation and classical Navier stokes equation was used to describe flow through reactor geometry. Convection-diffusion equations have been used to describe the mass transfer of the reactant species. The attained graphical illustrations derived from the simulation gave a clear view on the changing velocity and Reynolds number inside the FBR. The Reynolds number through the reactor scale was in a range of  *Re* = 200–370. The voidage of the fluidized area was fixed to 0.586 according to (1). Velocity profile inside the reactor showed to be in the range of 0.03–0.048 m/sec.
2. Reaction mechanism of phenol has been simplified taking into account the surrogate parameter TOC. The simplified reaction that represents phenol degradation took place in the defined fluidized area. The kinetic rate constants for reactions (3), (4), and (5) were found to be 0.0012 s^−1^, 6.35 × 10^−5^ M^−1^s^−1^, and 9.6 × 10^−7^ M^−1^s^−1^, respectively.
3. From the scale-up study it is shown that up to 3 times the size of the simulated reactor similarity on hydrodynamics and performance can be attained. This established procedure can be applied to other pollutant degradation for reactor systems similar to this work. In this way, prediction of final result can be done which can be a prominent tool in design and scale-up of reactor systems involving pollutant degradation.

Thus, the monitoring of the fluidization process can be done. Consequently, it can be concluded that application of CFD simulations for the fluidized bed catalytic reactor could address a better design or extrapolation of such water treatment devices and allow a better understanding of the physicochemical phenomena involved in water treatment processes. Therefore, the stated procedure is suitable for certain design of such fluidized bed reactors for predicting performance and scale-up.

## Figures and Tables

**Table 1:** Reactor description and applied conditions for CFD simulation.

Parameter	Specification
Operating temperature	298 K
Operating time	3600 sec
Solution density, *ρ*	1000 kg/m^3^
Solution viscosity, *μ*	0.008904 poise
[TOC]_in_	4.46 mol/m^3^ and 2.55 mol/m^3^
[H_2_O_2_]_in_	27 mol/m^3^–16 mol/m^3^
[Diffusion coefficient]_TOC_	1 × 10^−6^ m^2^/sec
[Diffusion coefficient]_OH_	1 × 10^−6^ m^2^/sec
[Fe^3+^] in reactor	1.54 and 0.88 gm/L
[Fe^2+^] in solution	14 and 8 mg/L
Amount of glass beads	30 gm/L
Reactor-dia.	7 cm
Reactor-height	70 cm
Reactor-volume	2 Liter
Operating flow rate	0.03 m/sec
Bed voidage, *ε*	0.586 (unit less)
Permeability, *κ*	5.9 m^2^

**Table 2:** Reactions and rate constants for complete mineralization of phenol by Fe^3+^/Fe^2+^/H_2_O_2_.

No.	Reaction	Rate constant	Reference
R1	Fe^3+^ + H_2_O_2_ → Fe^2+^ + H^+^ + HO_2_^∙^	1 × 10^−2^ M^−1^s^−1^	[20, 35]
R2	Fe^3+^ + HO_2_^∙^ → Fe^2+^ + H^+^ + O_2_	3.3 × 10^5^ M^−1^s^−1^	[36]
R3	Fe^2+^ + HO_2_^∙^ → Fe^2+^ + HO^∙^ + OH^−^	6.3 × 10^1^ M^−1^s^−1^	[35, 36]
R4	HO^∙^ + H_2_O_2_ → HO_2_^∙^ + H_2_O	3.3 × 10^7^ M^−1^s^−1^	[36]
R5	2HO_2_^∙^ → H_2_O_2_ + O_2_	8.3 × 10^5^ s^−1^	[37]
R6	2HO^∙^ → H_2_O_2_	4.2 × 10^9^ s^−1^	[35]
R7	HO^∙^ + phenol → 1,2-DHCD^∙^	3.3 × 10^9^ M^−1^s^−1^	[35]
R8	1,2-DHCD^∙^ + Fe^3+^ → Fe^2+^ + catechol	7.0 × 10^3^ M^−1^s^−1^	[35]
R9	1,4-DHCD^∙^ + Fe^3+^ → Fe^2+^ + hydroquinone	7.0 × 10^3^ M^−1^s^−1^	[35]
R10	1,2-DHCD^∙^ + HO^∙^ → THB	2.0 × 10^10^ M^−1^s^−1^	[35]
R11	Catechol + HO^∙^ → THCD^∙^	1.1 × 10^10^ M^−1^s^−1^	[20]
R12	THCD^∙^ + Fe^3+^ → THB + Fe^2+^ + H^+^	7.0 × 10^3^ M^−1^s^−1^	[35]
R13	Fe^3+^ + THB → Fe^2+^ + fumaric acid	1.0 × 10^1^ M^−1^s^−1^	[35]
R14	Fumaric acid + HO^∙^→ oxalic acid + CO_2_	6.0 × 10^9^ M^−1^s^−1^	[20]
R15	Oxalic acid + HO^∙^→ CO_2_	1.4 × 10^6^ M^−1^s^−1^	[20]
R16	Phenol + HO^∙^→ aromatics	0.3805 M^−1^s^−1^	[38]
R17	Phenol + HO^∙^→ muconic acid	1.933 × 10^−2^ M^−1^s^−1^	[38]
R18	Phenol + HO^∙^→ fumaric acid	1.5 × 10^−2^ M^−1^s^−1^	[39]
R19	H_2_O_2_→2HO^∙^	0.0012 s^−1^	From this study
R20	TOC_1_ + HO^∙^→ TOC_2_ + H_2_O	6.35 × 10^−5^ M^−1^s^−1^	From this study
R21	TOC_2_ + HO^∙^→ final product	9.6 × 10^−7^ M^−1^s^−1^	From this study

DHCD: di-hydroxy-cyclohexa-di-enyl radical; THB: tri-hydroxy-benzene; THCD: di-hydroxy-cyclohexa-di-enyl radical.

**Table 3:** CFD result and batch study result.

No.	Experimental condition	TOC conversion	
		Experimental study	Prediction by CFD for FBR-AOP
01	Phenol: 70 mg/L or TOC_in_: 4.46 mol/m^3^ H_2_O_2_: 980 mg/L	44%	48%

02	Phenol: 50 mg/L or TOC_in_: 2.55 mol/m^3^ H_2_O_2_: 560 mg/L	48%	46%

**Table 4:** Physical properties and operating conditions simulated reactor and potential reactors for scale-up.

Name	*m* value	Column Diameter, *D* (m)	Column Height, *H* (m)	Liquid density, *ρ*_*f*_ (kg/m^3^)	Liquid viscosity, *μ*_*f*_ (Pa*·*s)	Solid density, *ρ*_*s*_ (kg/m^3^)	Particle diameter, *d*_*p*_ (mm)	Minimum fluidization velocity, *U*_*mf*_ (m/s)	Initial liquid velocity, *U*_*O*_ (m/s)	Volume flow rate, *Q* (litre/min)
Simulated reactor	1	0.07	0.7	1000	0.0008	1600	2	0.02	0.05	0.61
RCTR1	1.5	0.105	1.05	1000	0.0008	1600	2	0.025	0.061	0.747
RCTR2	2	0.21	2.1	1000	0.0008	1600	2	0.035	0.087	1.057
RCTR3	2.5	0.5247	5.247	1000	0.0008	1600	2	0.055	0.137	1.671
RCTR4	3	1.575	15.75	1000	0.0008	1600	2	0.095	0.237	2.893
RCTR5	3.5	5.51	55.1	1000	0.0008	1600	2	0.18	0.444	5.413

**Table 5:** Dimensionless parameters corresponding to Table 4.

Tag name	$\frac{{U_{O}}^{2}}{gD}$	$\frac{\rho_{f}}{\rho_{s}}$	$\frac{U_{O}}{D}$	$\frac{d_{p}}{D}$	$\frac{\rho_{s}\rho_{f}{d_{p}}^{3}g}{{\mu_{f}}^{2}}$	$\frac{\rho_{f}U_{O}d_{p}}{\mu_{f}}$	$\frac{\rho_{f}U_{O}D}{\mu_{f}}$
Simulated reactor	0.003641	0.625	2.5	0.285714	20000	125	4375
RCTR1	0.003641	0.625	2.5	0.190476	20000	153.0931	8037.388
RCTR2	0.003641	0.625	2.5	0.095238	20000	216.5064	22733.17
RCTR3	0.003643	0.625	2.5	0.038117	20000	342.3266	89809.38
RCTR4	0.003641	0.625	2.5	0.012698	20000	592.9271	466930.1
RCTR5	0.003642	0.625	2.5	0.00363	20000	1109.265	3056025
